# Complete genome sequence and construction of an infectious full-length cDNA clone of a cucumber vein yellowing virus (CVYV) isolate from Portugal

**DOI:** 10.1007/s00705-021-05248-y

**Published:** 2021-10-04

**Authors:** K. Cordes, E. Maiss, S. Winter, H. Rose

**Affiliations:** 1 Institute of Horticultural Production Systems, Dept. Phytomedicine, Leibniz Universität Hannover, Herrenhäuser Str. 2, 30419 Hannover, Germany; 2 Leibniz Institute DSMZ, German Collection of Microorganisms and Cell Cultures, Inhoffenstraße 7 B, 38124 Braunschweig, Germany

## Abstract

Cucumber vein yellowing virus (CVYV) is a member of the genus *Ipomovirus* in the family *Potyviridae*. In the National Center for Biotechnology Information (NCBI) database, three complete genome sequences of CVYV isolates from Spain (NC_006941), Israel (KT276369), and Jordan (JF460793) are available. In this study, we report the complete sequence of an isolate of CVYV from Portugal (DSMZ PV-0776) along with the construction of an infectious full-length cDNA clone via Gibson assembly. The sequence of CVYV Portugal shows the closest relationship to a CVYV isolate from Spain (genome, 99.7% identity; polyprotein, 99.7% identity). The CVYV full-length cDNA clone was introduced by electroporation into *Rhizobium radiobacter* and infiltrated into the cotyledons of *Cucumis sativus* plantlets, resulting in symptoms resembling those of the wild-type virus. Transmission of the infectious CVYV full-length clone by the whitefly *Bemisia tabaci* was confirmed. This first report confirming the infectivity of a CVYV cDNA clone provides the opportunity to study gene functions in a consistent genomic background.

**Supplementary Information:**

The online version contains supplementary material available at 10.1007/s00705-021-05248-y.

Cucumber vein yellowing virus (CVYV) is a member of the genus *Ipomovirus* in the family *Potyviridae*. This genus includes seven species (ICTV Master Species List 2020.v1 [MSL36]). The first description of CVYV was published by Cohen and Nitzany in 1960 in Israel [1], and the virus has since been reported in the Middle East (Lebanon [2], Turkey [3], Jordan [4], Cyprus [5], and Iran [6]), Europe (Portugal [7], Spain [8], and France [9]) and Africa (Sudan [10] and Tunisia [11]). CVYV infects mainly plants in the family Cucurbitaceae, such as cultivated cucumber (*Cucumis sativus*), squash (*Cucurbita pepo*), melon (*Cucumis melo*), bottle gourd (*Lagenaria siceraria*), and watermelon (*Citrullus lanatus*) [12] as well as wild-growing cucurbits [13]. Symptoms are mainly described as yellowing of veins and leaves, stunting [1, 3], and, in the case of melons, fruit splitting [7]. Other experimental host plants that do not belong to the family Cucurbitaceae, including chervil (*Anthriscus cerefolium*, Apiaceae), tobacco (*Nicotiana clevelandii*, Solanaceae), tomato (*Solanum lycopersicum*, Solanaceae), and jimsonweed (*Datura stramonium*, Solanaceae) have also been reported to be infected [14]. In studies on weed species growing near or in greenhouses in Spain, the virus was also found in field bindweed (*Convolvulus arvensis*, Convolvulaceae), mallow (*Malva parviflora*, Malvaceae), and common sow thistle (*Sonchus oleraceus*, Asteraceae) [15]. In nature, CVYV is transmitted by the whitefly *Bemisia tabaci* in a semi-persistent manner, while the virus is also transmitted mechanically [16]. Like other potyvirids, ipomoviruses have a monopartite single-stranded positive-sense RNA genome, which has a genome-linked viral protein (VPg) covalently bound to the 5’ end and a polyadenylated 3’ end [17]. This genus is separated into three subgroups, according to their genome organization. In the N-terminal region, sweet potato mild mottle virus (SPMMV) [18] and tomato mild mottle virus (TMMoV) [19] contain P1 and HC-Pro; CVYV, squash vein yellowing virus (SqVYV) [20, 21] and coccinia mottle virus (CocMoV) [22] encode a duplicated P1 region (P1a and P1b); and cassava brown streak virus CBSV) and Ugandan cassava brown streak virus (UCBSV) have a P1 but neither a P1b nor an HC-Pro. The latter additionally encode a HAM1h domain between NIb and CP [23, 24]. Other proteins encoded are P3 (including PIPO [pretty interesting *Potyviridae* ORF]), 6K1, CI (cylindrical inclusion), 6K2, NIa-VPg (nuclear inclusion protein a–viral protein, genome-linked), NIa-Pro (nuclear inclusion protein a–protease), NIb (nuclear inclusion protein b, RNA-dependent RNA polymerase [RdRp]) and CP (coat protein) [17]. To date, there are three complete genome sequences of CVYV isolates available in the NCBI database: one from Spain (ALM32, NC_006941.1), one from Jordan (JF460793.1), and one from Israel (ISM, KT276369).

In this study, the complete genome sequence of an isolate of CVYV from Portugal (DSMZ PV-0776) was determined, and an infectious full-length cDNA clone was constructed. Sequence analysis revealed a high level of sequence identity (99.7%) when its nucleotide and encoded amino acid sequence were compared to those of CVYV from Spain (NC_006941.1).

At the Leibniz Institute DSMZ Plant Virus Department, cucumber plantlets (*C*. *sativus* cv. ‘Vorgebirgstraube’) that were naturally infected with CVYV PV-0776 served as the source of CVYV. The virus was further propagated in cucumber, and freshly infected plants were used to prepare dsRNA extracts at 15 days post-inoculation (dpi) [25]. Primers for the construction of a full-length cDNA clone were derived from the sequence of the isolate from Spain (NC_006941.1) and are listed in Supplementary Table S1. For the construction of an infectious full-length cDNA clone via Gibson assembly [26], the genome of CVYV was amplified as two fragments (without poly(A)) and integrated into the binary vector pDIVA (KX665539). A detailed cloning procedure is provided in the supplementary material. The cloned CVYV genome was sequenced and found to be 9734 nucleotides (nt) in length, with a large open reading frame extending from nt 68 to 9511 and terminated by a UGA stop codon (nt 9512-9414). Nucleotides 1-67 represent the 5’untranslated region (UTR) and nt 9515 to 9734, the 3’-UTR (without poly(A)). PIPO starts at nucleotide position 2989 with the conserved motif GA_7_ and ends at position 3228 with UAA, resulting in a protein of 77 amino acids (~9 kDa). When compared to the other available complete sequences, percent identity values for the complete genome/polyprotein are 99.7%/99.7% (vs. Spain), 94.3%/96.1% (vs. Israel), and 94.4%/96.1% (vs. Jordan), indicating the closest relationship to the isolate from Spain. In total, there are 36 nucleotide substitutions, resulting in eleven amino acid changes when compared to the isolate from Spain. The amino acid changes are located in P1a (four exchanges), P1b (two exchanges), NIa-Pro (two exchanges), and NIb (three exchanges). For detailed information, see Supplementary Table S2. A schematic representation of the genome of CVYV Portugal is shown in Figure 1.

**Fig. 1:**
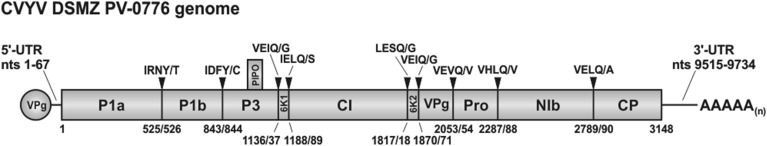
Schematic diagram of the genome organization of cucumber vein yellowing virus (CVYV) DSMZ PV-0776. Arrows indicate positions of cleavage sites. UTR, untranslated region; nts, nucleotides; aa, amino acids; PIPO, pretty interesting *Potyviridae* open reading frame; CI, cylindrical inclusion protein; VPg, viral protein, genome-linked; Pro, protease; NIb, nuclear inclusion protein b; CP, coat protein

The full-length cDNA clone was transformed into *Rhizobium radiobacter* GV2260 by electroporation and infiltrated into the lower surface of the cotyledons of four *C*. *sativus* cv. ‘Vorgebirgstraube’ plantlets using a needleless syringe. The OD_600_ in the inoculation buffer was adjusted to 1.0 ± 0.2. In all plants, symptoms occurred 19-21 dpi resembling those of the wild type (Fig. 2), and the virus was confirmed by specific RT-PCR. For detailed information, see “Infiltration of the full-length cDNA clone and verification of CVYV infection” in the supplementary material.

**Fig. 2:**
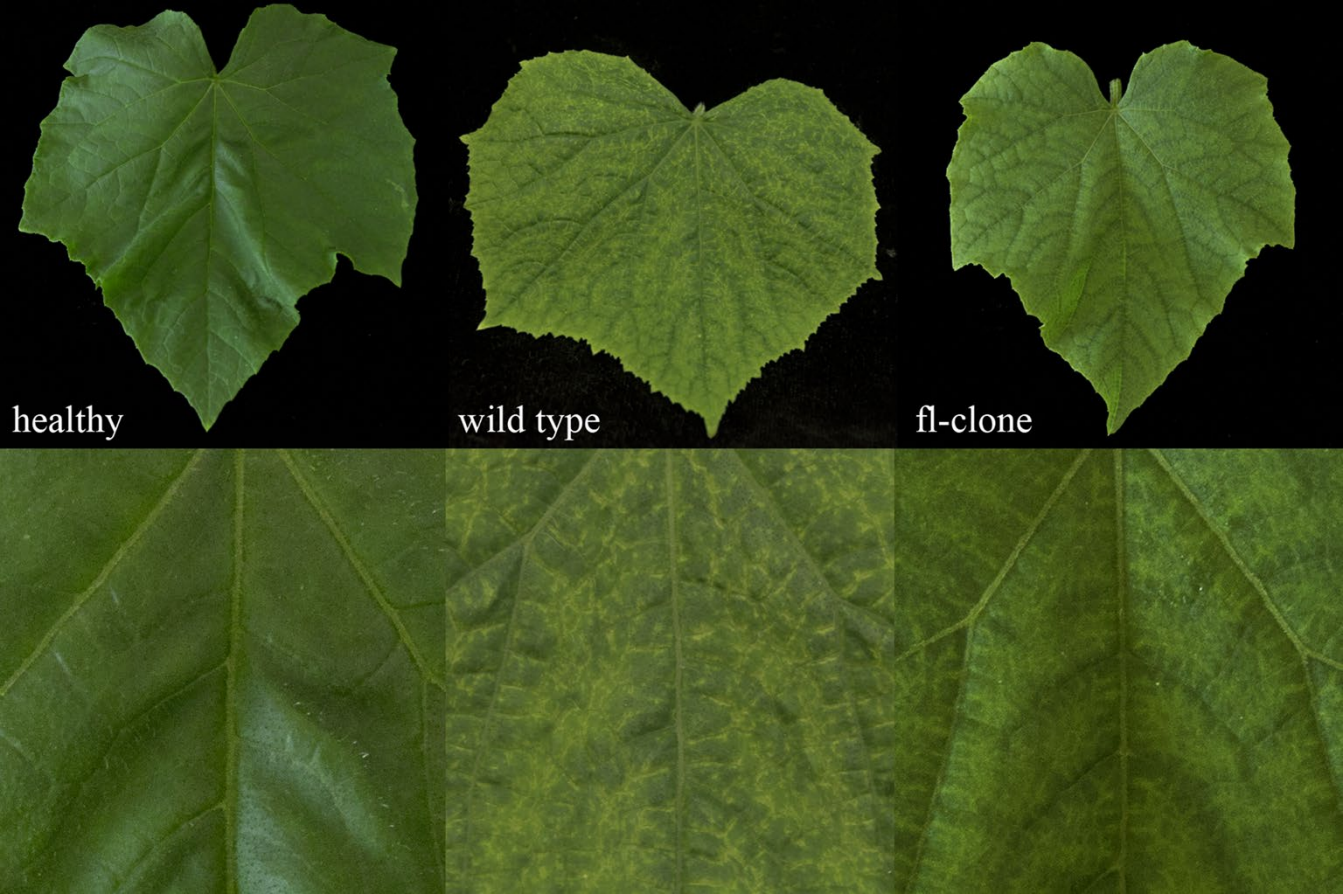
Symptom expression of cucumber vein yellowing virus (CVYV) DSMZ PV-0776 wild type-virus (16 dpi) and full-length cDNA clone (15 dpi) compared to an untreated healthy control in systemic leaves of *C*. *sativus* cv. ‘Vorgebirgstraube’. Top, whole leaves; bottom, detail

Semi-persistent transmission of the CVYV full-length clone by *B*. *tabaci* MEAM1 pol 63 [27] was confirmed at the Leibniz Institute DSMZ Plant Virus Department. The whiteflies (20-30 adults) were allowed to feed on infected donor plants (*C*. *sativus* cv. ‘Riesenschael’) for different lengths of time to determine the acquisition access and inoculation time. The CVYV full-length clone was readily transmitted. Efficient virus transmission was observed when whiteflies were kept overnight on infected plants. However, a two-hour acquisition/inoculation period was found to be sufficient for transmission of the CVYV full-length clone. Virus symptoms became noticeable 14 days after whitefly transmission and were similar to those of the wild-type virus isolate from Portugal.

CVYV Portugal is the first reported infectious and transmissible full-length cDNA clone of a virus isolate of the species *Cucumber vein yellowing virus*. This now provides the possibility to introduce genome modifications to study vital biological virus traits, transmission, symptomatology, and host range.

## Supplementary Information

Below is the link to the electronic supplementary material.

Supplementary file1 (FASTA 10 KB)

Supplementary file2 (FASTA 3 KB)

Supplementary file3 (PDF 350 KB)

## Data Availability

The manuscript has data included as electronic supplementary material.
