# Supplementary material for: Complete genome sequence and construction of an infectious full-length cDNA clone of a cucumber vein yellowing virus (CVYV) isolate from Portugal
Source: Arch Virol. 2021 Oct 4;166(12):3417–20. doi: 10.1007/s00705-021-05248-y (PMC8616879; doi:10.1007/s00705-021-05248-y)
Supplement: Supplementary file 3 — Supplementary file3 (PDF 350 KB) [file 705_2021_5248_MOESM3_ESM.pdf]

Complete genome sequence and construction of an infectious full-length cDNA clone of a cucumber vein yellowing virus (CVYV) isolate from Portugal

K. Cordes[1], E. Maiss[1], S. Winter[2], H. Rose[1]

[1] Institute of Horticultural Production Systems, Dept. Phytomedicine, Leibniz Universität Hannover, Herrenhäuser Str. 2, 30419, Hannover, Germany.

[2] Leibniz Institute DSMZ, German Collection of Microorganisms and Cell Cultures, Inhoffenstraße 7 B, 38124 Braunschweig, Germany

Corresponding author: Dr. Hanna Rose ORCID: 0000-0002-7226-1803

rose@ipp.uni-hannover.de

Construction of an infectious full-length clone of CVYV Portugal (DSMZ PV-0776)

Leaves of *C. sativus* systemically infected with cucumber vein yellowing virus (CVYV) DSMZ PV-0776 were homogenized in liquid nitrogen and stored at -80 °C. A dsRNA extraction of the freshly infected plants was performed 15 dpi [1]. Primers for the construction of an infectious full-length clone were derived from the sequence of the isolate from Spain (NC_006941.1). Full-length cDNA was produced by incubating 3 µl dsRNA as well as 1 µl of sense and antisense primer each (10 µM; salt free; Eurofins Genomics) for 5 min at 95 °C followed by a rapid cooling in liquid nitrogen. For detailed information on primers, see supplementary table S1. A 42 °C prewarmed reverse transcription mix containing 5 µl 5×RT Buffer (Thermo Fisher Scientific), 1 µl dNTPs (10 mM each; Thermo Fisher Scientific), 0.5 µl RevertAid Premium Reverse Transcriptase (200 U/µl; Thermo Fisher Scientific), 0.5 µl RiboLock (40 U/µl; Thermo Fisher Scientific) and 13 µl H_2O was added and the complete sample incubated at 42 °C for 1 h. For construction of an infectious full-length clone, the cDNA was amplified as two fragments being 4849 bp (fragment 1) and 4956 bp (fragment 2) in size (without poly(A)). The reactions were set up as follows: 2 µl cDNA, 10 µl 2× Phusion Flash High-Fidelity PCR Master Mix (Thermo Fisher Scientific), 1 µl primer sense and antisense each (10 µM, salt free, Eurofins Genomics) and 6 µl H_2O. The amplification was started with an initial denaturation at 98 °C for 15 s, followed by 34 cycles of 98 °C for 5 s, 53 °C for 5 s and 72 °C for 150 s and a final elongation of 5 min at 72 °C. After gel electrophoresis, the fragments were purified in a final volume of 20 µl (Quantum matrix solution, Bio-Rad). The ligation into the modified binary vector pDIVA (KX665539) was carried out via Gibson Assembly [2]. For this, 8 µl fragment 1 or fragment 2 were mixed with 2 µl of pDIVA vector, respectively and incubated at 50 °C for 1 h. After transformation into *Escherichia coli* NM522 [3], plasmids were isolated [4] and analysed via restriction analysis. This revealed six clones for pDIVA_fragment1 and ten clones for pDIVA_fragment2 that were pooled in a ratio 1:1 and diluted 1:50 in H_2O each before being used as a template in a subsequent PCR to generate the fragments, which were used for constructing the final clone. The larger fragment of 9134 bp contained the vector backbone plus fragment 1 and the smaller fragment was composed of fragment 2. Both reactions contained 1 µl respective diluted plasmid mix, 10 µl 2× Phusion Flash High-Fidelity PCR Master Mix (Thermo Fisher Scientific), 1 µl primer sense and antisense each (10 µM, salt free, Eurofins Genomics) and 7 µl H_2O. The amplification was performed as described above, only annealing temperature was set to 55 °C and elongation time to 120 s for fragment 1 inclusive pDIVA backbone and to 90 s for fragment 2. The resulting fragments were purified in 20 µl elution

buffer (SureClean Plus, Bioline) and treated as described before. One of the resulting plasmids was completely sequenced and tested for infectivity in *C. sativus*.

Infiltration of the full-length cDNA clone and verification of CVYV infection

The full-length cDNA clone was transformed into *R. radiobacter* GV2260 by applying an electric pulse of 1440 V for 6 ms to a mix of plasmid and cells [5, 6]. Subsequently 1 ml SOC medium was added and incubated for 3 h at 28 °C, after which 100 µl were plated on LB medium plus 50 µg/ml kanamycin. After an incubation period of about 36 h at 28 °C, five colonies were transferred to 15-20 ml liquid LB medium with 50 µg/ml kanamycin and incubated overnight at 28 °C on a shaker at 200 rpm. Bacteria were harvested by centrifugation for 5 min at 8000 rpm (Eppendorf MiniSpin, F45-12-11) and mixed with inoculation buffer (10 mM $MgCl_2$,10 mM MES, 100 µM acetosyringone, pH 5.2) up to an OD_{600}= 1.0 ±0.2. Infection was performed by infiltrating the suspension into the lower surface of the cotyledons of four *C. sativus* cv. 'Vorgebirgstraube' plantlets with a needleless syringe. In all plants, symptoms occurred 15-21 day post inoculation (dpi) resembling those of the wild type virus (Fig. 2). Presence of CVYV was confirmed by a specific RT-PCR in which a 543 nts fragment located at the 5'-end of the CP gene was amplified. Following total nucleic acids extraction [7], reverse transcription started by mixing 3 µl total nucleic acid extraction and 1 µl antisense primer (GCTGTTCTCATAGCCCAGTTTTCC, 10 µM, salt free, Eurofins Genomics) and incubating 3 min at 99 °C followed by a rapid cooling on ice. Then, 4 µl 5×RT Buffer (Thermo Fisher Scientific), 0.5 µl dNTPs (10 mM each; Thermo Fisher Scientific), 1 µl RevertAid Reverse Transcriptase (20 U/µl; Thermo Fisher Scientific) and 10.5 µl H_2O were added and the complete sample incubated at 42 °C for 1 h. The following PCR was composed of 2 µl cDNA, 5 µl 2× Phusion Flash High-Fidelity PCR Master Mix (Thermo Fisher Scientific), 1 µl primer sense GGCAGACGACATTGAGAAAGAAGC and antisense GCTGTTCTCATAGCCCAGTTTTCC each (10 µM, salt free, Eurofins Genomics) and 1 µl H_2O. The amplification was started with an initial denaturation at 98 °C for 10 s, followed by 30 cycles of 98 °C for 5 s, 55 °C for 5 s and 72 °C for 15 s and a final elongation of 5 min at 72 °C and revealed all plants to be infected.

Table S1: Oligonucleotides for the construction of the infectious full-length cDNA clone and verification of infection. Underlined parts are vector sequences.

**Use**	**Sequence**
Full-length cDNA	AGGAAGTTCATTTCATTTGGAGAGGAAATTACAAAACATTCGATCACATA<br>GAGATGCCATGCCGACCCTTTTTTTTTTTTTTTTTTTTT
Amplification of empty pDIVA for Gibson Assembly	CCTCTCCAAATGAAATGAACTTCCTTATATAG<br>GGGTCGGCATGGCATCTCCACCTCCTC
Amplification of fragment 1 for Gibson Assembly into pDIVA (template: full-length cDNA)	AGGAAGTTCATTTCATTTGGAGAGGAAATTACAAAACATTCGATCACATA<br>GAGATGCCATGCCGACCCACCCTATGTCAACATCATCAACCACTG
Amplification of fragment 2 for Gibson Assembly into pDIVA (template: full-length cDNA)	AGGAAGTTCATTTCATTTGGAGAGGCAGTGGTTGATGATGTTGACATAGGGT<br>GAGATGCCATGCCGACCCATAACTTTACGCATAAAGGCTAATATTCCAAG
Amplification of vector backbone and fragment 1 for Gibson Assembly (template: pDIVA_fragment 1)	GGGTCGGCATGGCATCTCCACCTCCTC<br>ACCCTATGTCAACATCATCAACCACTG
Amplification of fragment 2 for Gibson Assembly (template: pDIVA_fragment 2)	CAGTGGTTGATGATGTTGACATAGGGT<br>GAGATGCCATGCCGACCCATAACTTTACGCATAAAGGCTAATATTCCAAG
Verification of CVYV infection	GGCAGACGACATTGAGAAAGAAGC<br>GCTGTTCTCATAGCCCAGTTTTCC

Table S2: Nucleotide and amino acid exchanges between CVYV isolates from Portugal (MZ130935) and Spain (NC_006941.1). Nucleotide exchanges leading to amino acid exchanges are highlighted in bold, complete exchanged triplets are additionally underlined.

		Isolate				
Position		Portugal		Spain		
Genome	ORF	Nucleotide	Amino acid	Nucleotide	Amino acid	Gene/Protein
109	14	G	E	A	E	P1a
163	32	G	Q	A	Q	P1a
205	46	A	K	G	K	P1a
331	88	C	C	T	C	P1a
388	107	G	S	A	S	P1a
**479**	**138**	**G**	**A**	**C**	**R**	**P1a**
**480**	**138**	**C**	**A**	**G**	**R**	**P1a**
**481**	**138**	**G**	**A**	**C**	**R**	**P1a**
**482**	**139**	**A**	**T**	**G**	**G**	**P1a**
**483**	**139**	**C**	**T**	**G**	**G**	**P1a**
**484**	**139**	**T**	**T**	**G**	**G**	**P1a**
**494**	**143**	**A**	**N**	**T**	**Y**	**P1a**
**603**	**179**	**A**	**D**	**G**	**G**	**P1a**
652	195	G	K	A	K	P1a
754	229	G	K	A	K	P1a
1114	349	G	R	A	R	P1a
1318	417	T	T	C	T	P1a
1609	514	T	L	C	L	P1a
1678	537	C	Y	T	Y	P1b
**2001**	**645**	**T**	**F**	**C**	**S**	**P1b**
**2102**	**679**	**G**	**V**	**A**	**I**	**P1b**
2347	760	T	I	C	I	P1b
3268	1067	A	A	G	A	P3
3550	1161	C	D	T	D	6K1
3709	1214	G	Q	A	Q	CI
5743	1892	T	D	C	D	NIa-VPg
**6246**	**2060**	**T**	**V**	**C**	**A**	**NIa-Pro**
**6345**	**2093**	**A**	**Y**	**G**	**C**	**NIa-Pro**
**7074**	**2336**	**G**	**R**	**A**	**K**	**NIb**
7240	2391	C	D	T	D	NIb
7348	2427	G	T	A	T	NIb
**8033**	**2656**	**T**	**F**	**C**	**L**	**NIb**
**8217**	**2717**	**T**	**L**	**C**	**S**	**NIb**
8644	2859	C	D	T	D	CP
9530	-	C	-	A	-	3' UTR
9617	-	A	-	G	-	3' UTR

References

1. Morris TJ (1979) Isolation and analysis of double-stranded RNA from virus-infected plant and fungal tissue. Phytopathol 69:854
2. Gibson DG, Young L, Chuang R-Y et al. (2009) Enzymatic assembly of DNA molecules up to several hundred kilobases. Nat Methods 6:343–345
3. Hanahan D (1983) Studies on transformation of *Escherichia coli* with plasmids. J Mol Biol 166:557–580
4. Birnboim HC, Doly J (1979) A rapid alkaline extraction procedure for screening recombinant plasmid DNA. Nucleic Acids Res 7:1513–1523
5. Hellens R, Mullineaux P, Klee H (2000) Technical Focus: A guide to *Agrobacterium* binary Ti vectors. Trends Plant Sci 5:446–451
6. Mattanovich D, Ruker F, Machado AC et al. (1989) Efficient transformation of *Agrobacterium* spp. by electroporation. Nucleic Acids Res 17:6747
7. Menzel W, Jelkmann W, Maiss E (2002) Detection of four apple viruses by multiplex RT-PCR assays with coamplification of plant mRNA as internal control. J Virol Methods 99:81–92
